# Roles of Apolipoprotein E (ApoE) and Inducible Nitric Oxide Synthase (iNOS) in Inflammation and Apoptosis in Preeclampsia Pathogenesis and Progression

**DOI:** 10.1371/journal.pone.0058168

**Published:** 2013-03-05

**Authors:** Luyi Mao, Qiongjie Zhou, Shufeng Zhou, Rhonda R. Wilbur, Xiaotian Li

**Affiliations:** 1 Obstetrics and Gynecology Hospital, Fudan University, Shanghai, China; 2 Department of Pharmaceutical Sciences, College of Pharmacy, University of South Florida, Tampa, Florida, United States of America; 3 Institute of Biomedical Sciences, Fudan University, Shanghai, China; 4 Shanghai Key Laboratory of Female Reproductive Endocrine Related Diseases, Shanghai, China; Van Andel Institute, United States of America

## Abstract

**Objectives:**

To investigate potential roles of inducible nitric oxide synthase (iNOS) and apolipoprotein (apoE) in inflammation and apoptosis promoting pathological changes in preeclampsia in pregnant mice with apoE and/or iNOS knock out.

**Methods:**

B6.129 mice were crossed to produce WT, apoE^−/−^, apoE^+/−^, iNOS^−/−^, iNOS^+/−^ and apoE^−/−^iNOS^−/−^ groups. Variants were confirmed by PCR. Serum lipid parameters (triglycerides, TG; total cholesterol, TC; high density lipoprotein, HDL; and low density lipoprotein, LDL), NO levels and placental electronic microscopic ultrastructures were evaluated, and blood pressure (BP), 24-hour urine protein and pregnancy outcomes were recorded for pregnant F1 generation mice. Placental expressions of inflammatory (tumor necrosis factor-α, TNF-α; interleukin-6, IL-6; nuclear factor-κB, NF-κb) and apoptotic markers (Bcl-2 associated X protein, Bax, B-cell lymphoma/leukemia-2, Bcl-2, and Caspase-3) were evaluated via Western blot.

**Results:**

Serum lipids, BP and 24-hour urine protein levels were shown to be significantly higher and parturition and placenta weights were lower in apoE^−/−^ and apoE^−/−^iNOS^−/−^ groups (p<0.05). NO levels were lower in the apoE^−/−^iNOS^−/−^ group. In addition, inflammatory/apoptosis parameters, including TNF-α, IL-6, NF-κb, Bax, Bcl-2 and Caspase-3 in the apoE^−/−^iNOS^−/−^ group (p<0.01), as well as in the apoE^−/−^ group (p<0.05), and NF-κB, Bax in iNOS^−/−^ group (p<0.05) were higher compared with WT group. However, most of the inflammatory/apoptosis parameters in the iNOS^+/−^ and the apoE^+/−^ groups (p>0.05) showed no differences. In addition, placenta vascular endothelial and trophoblast cell morphological changes were demonstrated in both the apoE^−/−^iNOS^−/−^ and apoE^−/−^ groups.

**Conclusion:**

Elevated lipid metabolism and inflammatory/apoptosis parameters suggest a potentially significant role of apoE in preeclampsia pathology, as well as a relationship between iNOS and preeclampsia progression.

## Introduction

Preeclampsia is one of the principal causes of maternal morbidity and mortality in developing countries [1], characterized by elevated maternal blood pressure and proteinuria [2]. Placental dysfunction caused by endothelial injury [3] and inflammatory responses [4], [5] is regarded as a primary cause of preeclampsia. However, specific mechanisms remain unclear [1].

Preeclampsia is associated with abnormal lipid metabolism [6], [7], which promotes the inflammatory response [5], [8], [9]. As an important component of the reverse cholesterol transport pathway, apoE is an essential ligand for the uptake and clearance of atherogenic lipoproteins [10], [11]. ApoE plays an important role in atherosclerosis by modifying inflammatory responses and facilitating cholesterol efflux from foam cells [12]. Study has demonstrated that preeclampsia is characterized by profound lipid abnormalities and acute atherosis in placental bed similar to those present in atherosclerosis [13]. In addition, atherosclerosis is often seen in apoE^−/−^ mice [14]. And apoE polymorphism in women may be a risk factor for preeclampsia [15]. Different lines of evidence indicated that abnormal lipid metabolism is not merely a manifestation of preeclampsia, but that it is directly involved in its pathogenesis [16]. However, not all women with hyperlipidemia automatically react with increases in blood pressure and proteinuria during pregnancy. Therefore, there remains unsolved problems which have to be explored.

It is well known that nitric oxide (NO) plays an essential role in the maintenance of blood pressure and the development of preeclampsia. It is synthesized from the nonessential amino acid L-arginine by the action of NOS. There are three isoforms of NOS including nNOS (neuronal), iNOS (inducible) and eNOS (endothelial), in which nNOS and eNOS are considered as constitutive NOS (cNOS), producing small basal levels of NO and stimulated via calcium/calmodulin. iNOS is mainly produced in macrophage cells and is normally present in only very small quantities unless promoted by pro-inflammatory molecules, bacterial products or infection [17], [18]. INOS has a higher NO synthesis capacity compared with cNOS [19]. And it has been detected that iNOS mediated NO production can lower blood pressure in situations of systemic inflammatory reactions like sepsis [20]. iNOS expression has also been detected in placental tissue(within the placenta, iNOS is localized in villous stroma and in extravillous trophoblast [21]) from normal and hypertensive pregnancies [22], [23], [24]. However, the relationship between iNOS and preeclampsia is still controversial [25]. Since inflammatory cytokines and NO have been suspected to be important contributors in the pathologic process of preeclampsia [26] [27], [28],it is interesting to access whether iNOS is associated with the pathogenesis of preeclampsia, particularly in the mechanism of disorders of pregnancy caused by dyslipidemia.

So we proposed that the dyslipidemia related to the apoE may be associated with the pathogenesis of preeclampisa, and iNOS may be involved in the mechanism. In this study apoE and iNOS knockout (KO) mice models were established and verified. Moreover, pregnancy outcomes, inflammatory and apoptosis biomarkers were evaluated to determine the potential effects of apoE and iNOS, as well as alteration of iNOS activity secondary to apoE deficiency in preeclampsia.

## Materials and Methods

### 1. Animals

ApoE^−/−^ mice and iNOS^−/−^ mice were purchased from the Model Animal Research Center MARC at Nanjing University (body weight 25–30 g, 8–12 weeks old) and housed individually under 18–28°C, 40–70% humidity and regulated lighting (12-hour light/dark cycle). ApoE^+/−^ mice were produced by crossing wild type (WT) mice with apoE^−/−^ mice, and iNOS^+/−^ by crossing WT mice with iNOS^−/−^ mice. The double knockout (dko) (apoE^−/−^iNOS^−/−^) lineage was produced by six cross backs performed by the Fudan University Animal Care Committee. The breeding protocol provided a six group murine model: (1)WT; (2)apoE^−/−^; (3)apoE^+/−^; (4)iNOS^−/−^; (5)iNOS^+/−^; and (6)apoE^−/−^iNOS^−/−^. And the number of the mice in each group was 7. All procedures were approved by the Fudan University Health Science Center Animal Ethics Committee.

### 2. Genotype Identification by PCR Assay

Tail biopsies were obtained at 4 weeks age and total genomic DNA was isolated by DNAzol™ solution. DNA was amplified by PCR for sequences in exon12, exon 13 and Neo insert in iNOS^−/+^ (108 pb and 275 pb) containing mouse lines, and Neo insert in iNOS^−/−^ (275 pb). The following primers for iNOS genotyping were included: (1) 5′-ACATGCAGAATGAGTACCGG-3′(oMIR1216); (2) 5′-TCAACATCTCCTGGTGGAAC-3′(oMIR1217); (3) 5′-AATATGCGAAGTGGACCTCG-3′(oMIR1218), as described by Jackson laboratory. DNA was amplified by PCR for sequences in exon 3 and Neo of 155 pb, 255 pb for insert in apoE^+/−^ containing mouse lines, and PCR sequences of Neo 255 bp for apoE^−/−^. Primers for apoE^−/−^ genotyping were the following: (1)5′-GCCTAGCCGAGGGAGAGCCG-3′(oMIR0180), (2)5′-TGTGACTTGGGAGCTCTGCAGC-3′(oMIR0181); (3) 5′-GCCGCCCCGACTGCATCT-3′(oMIR0182), as described by Jackson laboratory.(iNOS:geneID18126; apoE: geneID11816).

### 3. Blood Pressure, Urine Protein and Parturition and Placenta Weights Detection

Day 0 of pregnancy was determined by observation of a vaginal plug. Systolic BP during pregnancy was recorded by non-invasive measurement (BP-98A, Softron, JAPAN) every 4 days. Placentas were harvested at parturition and stored at −80°C. Blood samples were obtained by ocular enucleation under sodium pentobarbitone anesthesia on day 19 of pregnancy. Pregnant mice were housed in individual metabolic cages with free access to food and water. 24-hour urine samples were collected, stored at –80°C, and analyzed via pyrogallol red kit (DiaSys Diagnostic Systems GmbH, GERMANY).

### 4. Serum Lipids Levels and NO Level Measurements

The serum TG, TC, HDL and LDL were measured using Hitachi7600 Automatic Biochemistry Analyzer. Serum and placental NO levels were evaluated using nitrate enzyme reduction method via NO kit (A013-1) provided by Nanjing Jiancheng Bioengineering Institute. Blood and placenta samples were taken into tubes and stored at −80°C. Metabolites of NO were measured using Griess reagent, by commercially available kit. Briefly, aliquots of serum and placentas in duplicate were incubated in room temperature with enzyme cofactors and nitrate reductase for 1 h to convert NO_3_ to NO_2_. Total NO_2_ was then analysed by reacting the samples with 50µL of Griess reagent,NO_2_ in serum and placenta were estimated from a standard curve measuring the absorbance at 540 nm [29].

### 5. Electron Microscopy

Placentas were obtained at parturition, fixed in 2.5% glutaraldehyde in 0.1 M phosphate buffer (PB), post-fixed in 1.0% osmium tetroxide and embedded in Epon 812. Semi-thin sections (1 Am) were stained with toluidine blue (TB) and examined microscopically. Ultrathin sections were double stained with uranyl acetate and lead citrate. Placental ultrastructure was observed via JEM-1200EX electron microscopy (JEOL, Tokyo, JAPAN).

### 6. Placental Expression of Inflammatory and Apoptosis Factors by Western Blot Assay

Placentas were homogenized in 2 mL of TPER Tissue Protein Extraction Reagent (310003 BioonGroup Corporation, Shanghai, CHINA) incubated on ice for 7 minutes and centrifuged at 10,000 g for 10 minutes at 4°C. Samples were run through a 4% stacking and 8% separating polyacrylamide gel for 60 minutes. Electrophoresis of 100 Ag of the total protein was performed on 10% SDS-polyacrylamide gels and the protein was transferred to a nitrocellulose membrane (Boster, Huhan, CHINA). Membranes were blocked for one hour at room temperature in 10% skimmed milk powder, incubated for one hour at room temperature in a solution containing 1∶1000 monoclonal mouseβ-actin (Boster, Huhan, CHINA) and 1∶1000 Bax, Bcl-2, caspase-3, IL-6, TNF-αand NF-κB antibodies,washed 4 times for 5 minutes each in filter-sterilized PBS containing 0.1% Tween-20 at room temperature, and incubated in a solution containing 1∶2000 sheep anti-rabbit IgG antibody labelled with HRP secondary antibodies (Santa Cruz Co.Ltd). Both total protein gels and anti-IL-6, -TNF-a, -NF-κB, -Bcl-2, -Bax, and caspase-3 blotted nitrocellulose membranes were visualized by Molecular Analyst software.

### 7. Statistical Analysis

Data were presented as mean±SD. Statistical analysis was performed using SPSS statistical software version 14.0 (SPSS, System for Windows, Chicago, IL, USA). Comparisons were performed with the use of Friedman rank-sum test. When a significant difference was detected, multiple-comparison analysis was performed using the Least Significant Difference (LSD) test after the rank transformation of the data. [30], [31]. P<0.05 was considered statistically significant.

## Results

### 1. Genotypic Determination, Serum Lipid Concentrations and NO Levels in Pregnant Mice

**Figure 1:**
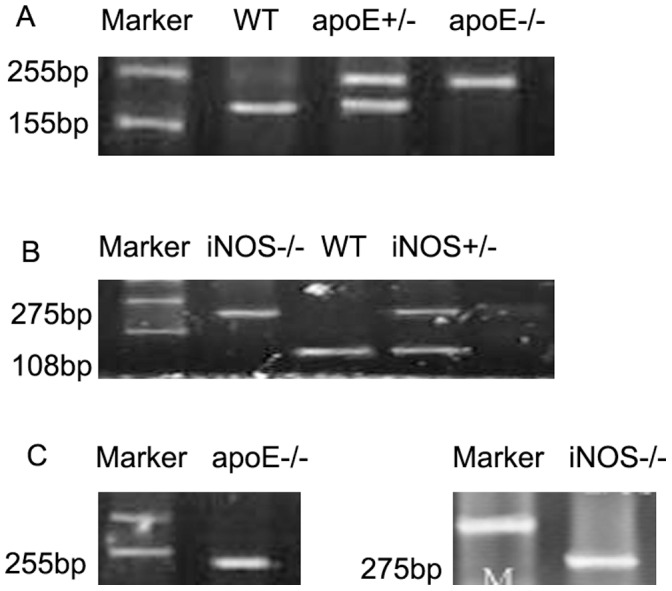
Genotypic determination by PCR. (**A**) apoE^−/−^, apoE^+/−^ and WT groups; (**B**) iNOS^−/−^,iNOS^+/−^ and WT groups; (**C**) genotype of apoE^−/−^iNOS^−/−^ group.

PCR results showed a specific band of 155 bp in the WT group, 255 pb in the ApoE^−/−^ group, 155 pb and 255 pb bands in the apoE^+/−^ group, 275 pb in the iNOS^−/−^ group, 108 pb and 275 pb in the iNOS^+/−^ group, and 255 pb and 275 pb bands in the apoE^−/−^iNOS^−/−^ group (Fig. 1).

Compared with the WT group, serum TG, TC and LDL levels were significantly higher and HDL level was lower in apoE^−/−^ and apoE^−/−^iNOS^−/−^ groups at the third trimester (p<0.05),while these parameters remained unchanged in apoE^+/−^, iNOS^−/−^ and iNOS^+/−^ groups, with the exception of a higher TC and lower HDL in the apoE^+/−^ group, compared with WT mice (p>0.05). Furthermore serum TG, TC and LDL levels were higher and HDL levels were lower in apoE^−/−^iNOS^−/−^ group compared with those in the apoE^−/−^ group (p<0.05) (Table 1).

**Table 1:** Comparison of serum lipid and NO levels in pregnant F1 generation mice during the third trimester of pregnancy.

	WT(n = 7)	apoE^−/−^(n = 7)	apoE^+/−^(n = 7)	iNOS^−/−^(n = 7)	iNOS^+/−^(n = 7)	apoE^−/−^I OS^−/−^(n = 7)
**TG(mmol/L)**	0.231±0.055	0.401±0.097*	0.253±0.038	0.387±0.199	0.252±0.239	0.711±0.134** ^#^
**TC(mmol/L)**	1.813±0.520	6.025±1.112**	3.571±0.394*	1.512±0.823	1.91±0.426	7.624±1.233** ^#^
**LDL(mmol/L)**	0.985±0.455	1.758±0.084*	1.045±0.349	0.851±0.325	0.766±0.244	2.904±0.686** ^#^
**HDL(mmol/L)**	0.621±0.296	0.121±0.301**	0.400±0.025*	0.547±0.172	0.667±0.354	0.284±0.621** ^#^
**Serum NO (µmol/L)**	24.5±2.2	23.8±2.4	24.00±2.0	23.1±3.7	26.6±4.3	14.2±1.6** ^#^
**Placental NO(µmol/g)**	22.8±2.5	19.2±3.1*	27.44±4.32	20.4±2.9	23.3±4.1	10.5±1.9** ^#^

Data is presented as mean±SD.

* p<0.05 when compared with the WT group,

** p<0.001 when compared with the WT group, and ^#^p<0.05 when the apoE^−/−^iNOS^−/−^ group was compared with apoE^−/−^ group.

In addition, a lower placental NO level was demonstrated in the apoE^−/−^ group, and serum and placental NO level in the apoE^−/−^iNOS^−/−^ group were noted compared with the WT group (p<0.05). Furthermore, serum and placental NO levels were much lower in the apoE^−/−^iNOS^−/−^ group compared with those in the apoE^−/−^ group (p<0.05). There was no difference in NO levels between apoE^+/−^, iNOS^−/−^, iNOS^+/−^ groups and WT group (p>0.05) (Table 1).

### 2. Blood Pressure, Urine Protein, Birth Weight and Placenta Characteristics in F1 Generation Mice

**Figure 2:**
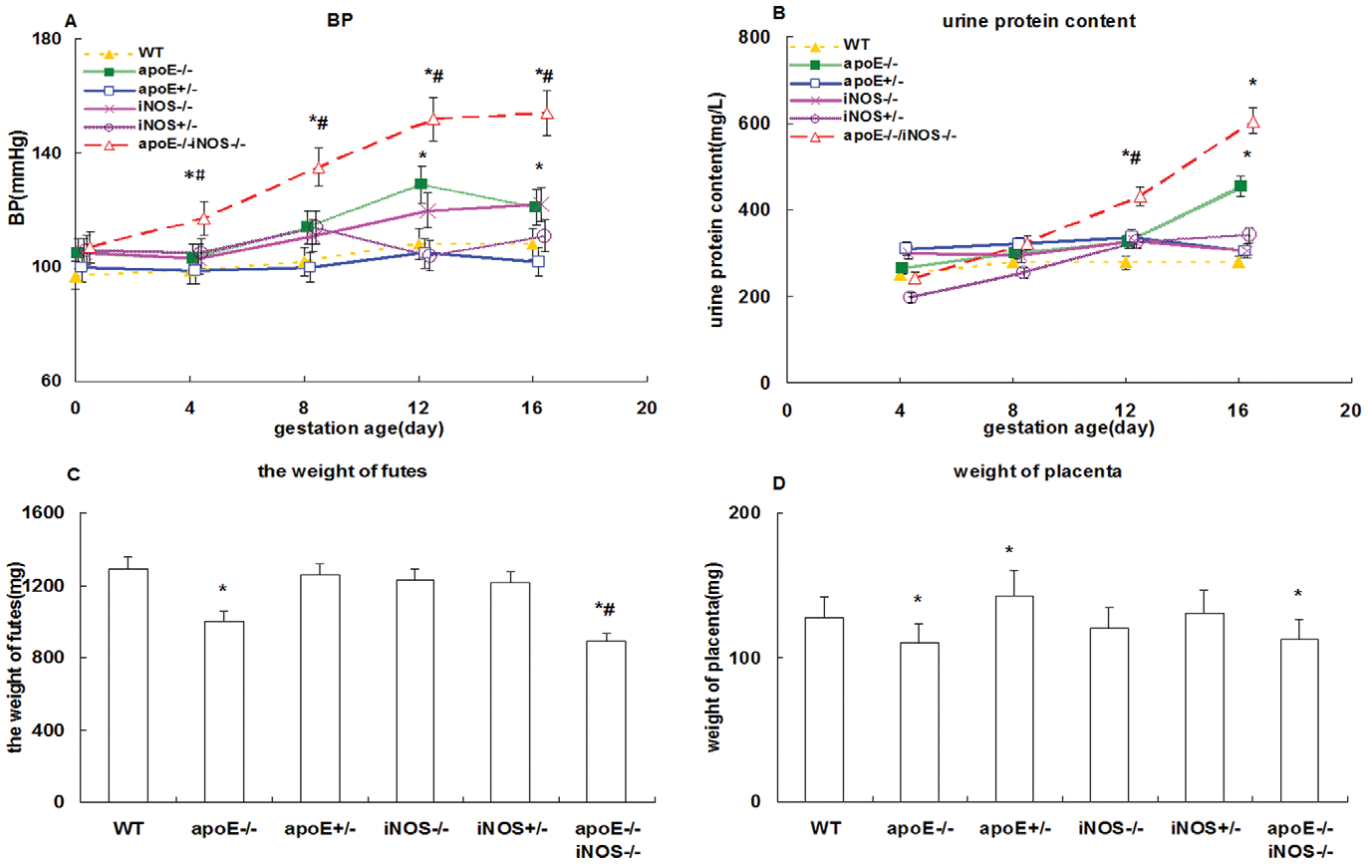
Blood pressure, urine protein, birth weight and placenta characteristics in F1 generation mice (A) BP during gestation; (B) Urinary protein during pregnancy; (C) Parturition weight (at delivery); (D) Placenta weight. *p<0.05 when compared with WT group, ^#^p<0.05 when apoE^−/−^ group compared with apoE^−/−^iNOS^−/−^.

During the course of pregnancy, blood pressure (BP) in apoE^−/−^ and apoE^−/−^iNOS^−/−^ groups significantly increased (p<0.05) compared with the WT group. The difference of BP between the apoE and WT increased with the gestational age, particularly after 12th day of gestation. The apoE^−/−^iNOS^−/−^ group demonstrated BP was significantly higher compared with the apoE^−/−^ group from 4th day of gestation (p<0.05) (Fig. 2A). Similar changes were also found in the urine protein of apoE^−/−^ and apoE^−/−^iNOS^−/−^ groups from the 12th day of gestation(p<0.05) (Fig. 2B).The differences of BP and urine protein between apoE^+/−^, iNOS^−/−^, iNOS^+/−^ groups and WT group were not significant (p>0.05).

Parturition and placenta weights were lower in apoE^−/−^ (parturition weight 1004±130 mg and placenta weight 110±7.2 mg) and apoE^−/−^iNOS^−/−^ groups (parturition weight 891±31 mg and placenta weight112±6.0 mg) (p<0.05). However, there was no difference between other groups and WT group (parturition weight 1289±83 mg and placenta weight126±13 mg) (p>0.05) (Fig. 2C, D).

**Figure 3:**
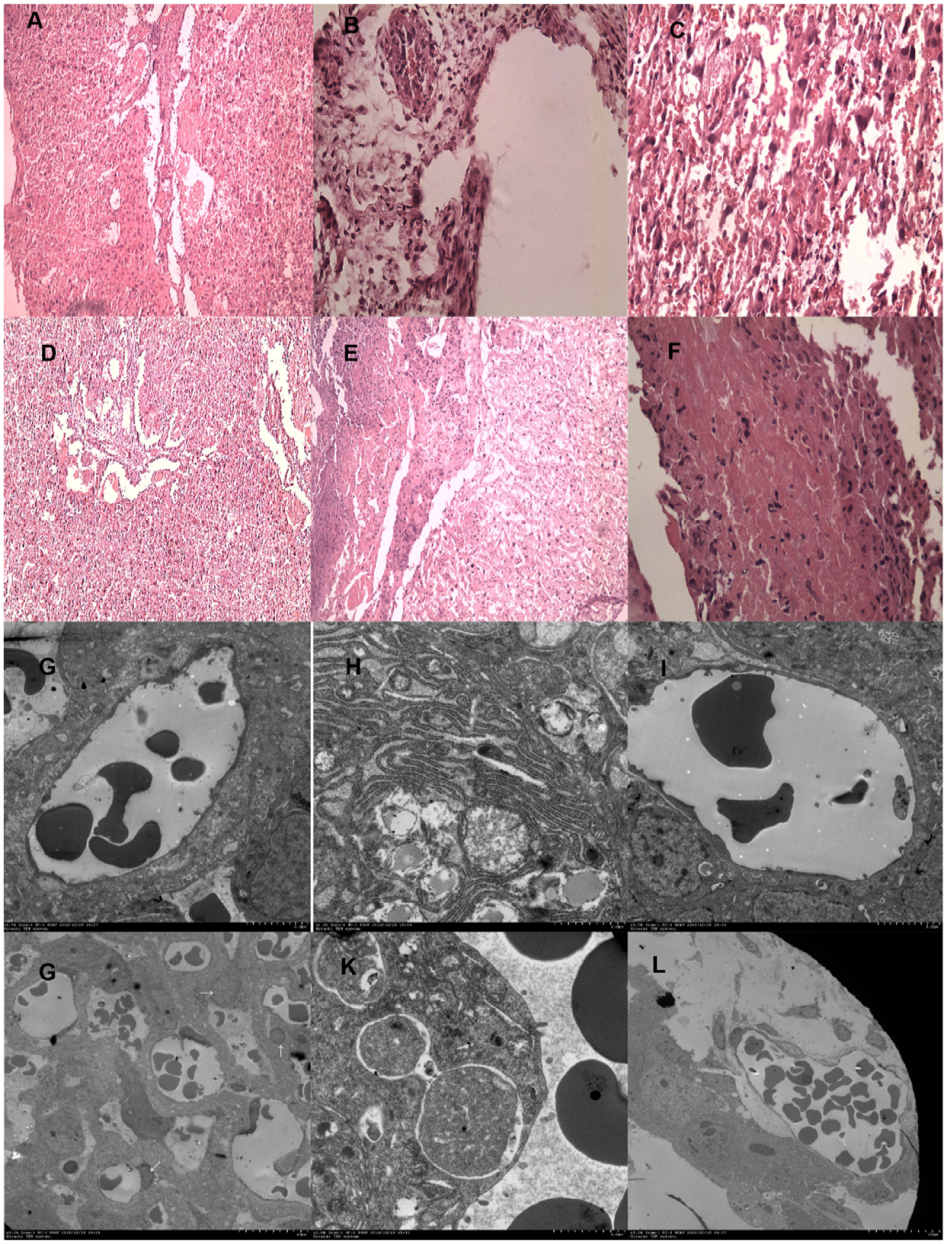
Placenta structure characteristics determined by electron microscopy and HE staining. Placenta ultrastructure×2000; HE×40.(**A**) placenta structure of WT mice by HE staining; (**B**) placenta structure of apoE^−/−^ mice by HE staining; (**C**) placenta structure of apoE^+/−^ mice by HE staining; (**D**) placenta structure of iNOS^−/−^ mice by HE staining; (**E**)placenta structure of iNOS^+/−^ mice by HE staining; (**F**) placenta structure of apoE^−/−^iNOS^−/−^ mice by HE staining; (**G**) placenta structure of WT mice by electron microscopy; (**H**) placenta structure of apoE^−/−^ mice by electron microscopy; (**I**) placenta structure of apoE^+/−^ mice by electron microscopy; (**J**) placenta structure of iNOS^−/−^ mice by electron microscopy; (**K**) placenta structure of iNOS^+/−^ mice by electron microscopy; (**L**) placenta structure of apoE^−/−^iNOS^−/−^ mice by electron microscopy.

Electron microscopy ultrastructure detection showed morphological changes in apoE^−/−^ and apoE^−/−^iNOS^−/−^ groups, including: (1) nuclei became heteromorphic; (2) nuclear membranes showed serrate pyknosis; (3) disappearance of nuclear membranes; (4) missing and abnormal chromosomal distribution in placental villus; (5) vascular endothelial and trophoblast cells suggesting potential apoptosis; (6) placental villus interstitial edema surrounding the blood vessels; (7) intracytoplasm membranous structure regression in various patterns; (8) blurry and irregular mitochondrial crista with pale mitochondrial stroma and nucleus edema; (9) morphological changes indicating apoptosis and vacuolization in vascular endothelial and trophoblast cells, including expanded hyperplasia endoplasmic reticulum and fat vesicle deposition. In addition, placental capillary hyperplasia aggregation at the periphery of the nucleons was seen in the apoE^−/−^ group and capillary hyperplasia was observed in iNOS^−/−^ group. However, no ultrastructural changes were seen in apoE^+/−^, iNOS^+/−^ and WT groups. HE staining demonstrated similar characteristics, with heteromorphic nuclei in apoE^−/−^ and apoE^−/−^iNOS^−/−^ groups (Fig. 3).

### 3. Inflammatory and Apoptotic Biomarker Detection via Western Blot

**Figure 4:**
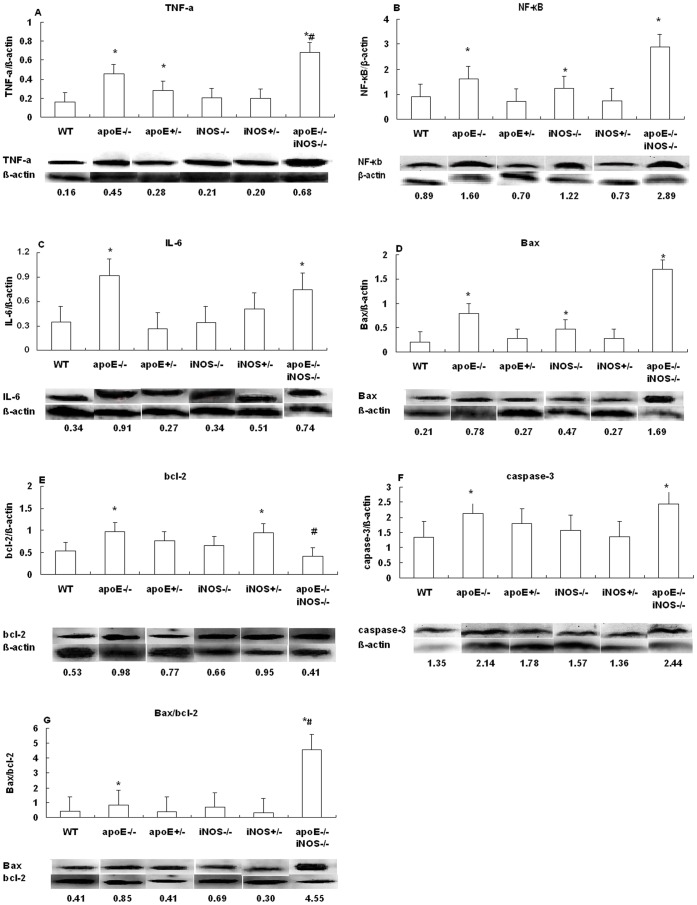
Placenta expression of inflammatory and apoptosis biomarkers determined by Western blot. (**A**) TNF-α; (**B**) NF-κb; (**C**) IL-6; (**D**) Bax; (**E**) Bcl-2; (**F**) Caspase-3; (**G**) Bax/Bcl-2. Data is presented as mean±SD. ^*^p<0.05 when compared with the WT group, ^#^p<0.05 when the apoE^−/−^ group was compared with apoE^−/−^iNOS^−/−^.

Placental inflammatory markers, including IL-6, TNF-α and NF-κB expressions were higher in the apoE^−/−^ group compared with the WT, and a much higher level of TNF-α was demonstrated in apoE^−/−^iNOS^−/−^ group (p<0.05). No significant changes were shown between apoE^+/−^, iNOS^−/−^, iNOS^+/−^ groups and WT group (p>0.05), with the exception of increased NF-κB in the iNOS^−/−^,and TNF-α in apoE^+/−^ group (p<0.05) (Fig. 4A, B, C).

Placenta apoptosis biomarkers in the apoE^−/−^iNOS^−/−^ group were higher than those in the WT group, including a 6-fold increase in Bax (1.692±0.214, p<0.001), a 1.7-fold increase in caspase-3 (2.438±0.520, p<0.001) and a 10-fold increase in Bax/Bcl-2 (4.409±1.580; p<0.001), and a similar characteristic of higher expression level of apoptosis biomarkers were found in the apoE^−/−^ group. No significant difference of expressions was observed between apoE^+/−^, iNOS^−/−^, iNOS^+/−^ groups and WT group (p>0.05), except the higher levels of Bax in the iNOS^−/−^ and Bcl-2 in iNOS^+/−^ group (p<0.05) (Fig. 4D, E, F, G).

## Discussion

In this study we established murine apoE and iNOS KO models and investigated their potential roles in apoptosis and inflammatory process of preeclampsia pathology using Western blot. Our study suggested that apoE and iNOS may offer placental protection in women with preeclampsia.

Preeclampsia-like syndromes, including higher BP, increased urine protein and decreased parturition and placental weights, were observed in apoE^−/−^ mice. Hyperlipidemia (higher levels of serum TG, TC and LDL and lower level of HDL), increased inflammation reaction (increasing levels of IL-6, TNF-αand NF-κB) and apoptosis (higher levels of caspase-3, Bax, higher ratio of Bax/Bcl-2 and histologic evidence) in placentas were found in apoE^−/−^ mice as well. Similar results were obtained in the research that up-regulation of pro-inflammatory cytokines like TNF-a, IL-6 and IL-12 were found in patients with dyslipidemia and in animal investigation of apoE^−/−^ mice [32], [33], [34], [35]. A generalized systemic maternal inflammatory response in preeclampsia [36] with increasing serum levels of TNF-α [37]and IL-6 [38], was thought to be caused by secretion of up-regulated inflammatory mediators from disordered placenta [37], [39], [40], so we focused on the inflammatory factors in placenta. In addition, potential apoptosis in apoE^−/−^ mice provided evidence that the deficit of apoE may lead to placental apoptosis contributing to preeclampsia, supporting previous works by Reckless and Chen in the liver of apoE^−/−^ mice [41], [42]. Thus, the dyslipismia of apoE^−/−^ may present as a pathogenic cause in inflammatory response and apoptosis of preeclampsia.

In our experiment no changes of proteinuria, blood pressure or NO level in serum and placentas were observed in iNOS^−/−^ mice compared with WT mice, which was in agreement with the study of Edward showing that the lack of iNOS cannot result in the change of the blood pressure [43]. A possible explanation could be that cNOS(eNOS and nNOS) is considered as the primary source of NO which plays an important role in the regulation of systemic blood pressure, blood flow and regional vascular tone in physiological condition [25], [44]. Without being induced by pro-inflammatory molecules, the single deletion of iNOS may not have effect on the amount of NO production.

Since not all women with hyperlipidemia result in preeclampsia during pregnancy, another factor may be involved in the mechanism in the hyperlipidemia-related disorder during pregnancy. Our data showed, compared with apoE^−/−^ mice, more significant preeclampsia-like syndromes, remarkable inflammation reaction and apoptosis in placentas were found in apoE and iNOS dko mice accompanied with decline of NO concentration of serum and placenta. Previous study showed that in response to vascular injury, iNOS produced higher levels of NO than eNOS [45]. Our study suggested that the absence of iNOS failed to respond to the inflammation caused by the deletion of apoE and resulted in imbalance of systemic and local NO production. And NO produced by iNOS played a protective role during acute inflammatory injury and in apoptosis [46], [47], [48]. The clinical studies of Tranquilli et al in that severe hypertensive disorders, such as HELLP syndrome, have been associated with reduced iNOS mRNA level [49] or protein abundance [24] compared with normal pregnancies. Therefore, it can be inferred that, the dysfunction of iNOS may be involved in the mechanism of PE progression caused by hyperlipidemia.

In summary, the possible pathogenesis of preeclampisa is inflammation caused by the dyslipidemia through the knockout of apoE, and the dysfunction of iNOS could contribute to exacerbation of preeclampsia.
